# Older HIV-infected individuals present late and have a higher mortality: a UK clinic cohort study

**DOI:** 10.1186/1758-2652-13-S4-P167

**Published:** 2010-11-08

**Authors:** CC Iwuji, D Pao, Y Gilleece, M Fisher, D Churchill

**Affiliations:** 1 Royal Sussex County Hospital, Dept of HIV/GUM, Brighton, UK; 2 Brighton and Sussex University Hospitals, Dept of HIV/GUM, Brighton, UK

## Introduction

Current UK guidelines recommend initiation of HAART in all patients with a CD4 count of <350 cells/mm^3^. The success of this policy depends on early HIV diagnosis. One-third of individuals in the UK have a CD4 cell count <200 cells/mm^3^ at diagnosis.

According to the European consensus, late presentation for care refers to persons presenting with CD4 cell count <350 cells/mm^3^ or presenting with an AIDS-defining event, regardless of the CD4 count.

## Purpose of the study

Applying only the current CD4 definition for late presentation, we examined the influence of age and calendar period on stage of HIV presentation and the impact of late presentation on mortality.

## Methods

Data were collected on patients diagnosed with HIV infection from 1st January 1996 to May 2010. Age was studied as a binary exposure variable (< 50 versus > 50 years). Patients were further stratified by time of diagnosis into 1996-2001, 2002-2005 and 2006-2010 periods. Logistic regression modelling was used to estimate the likelihood of late presentation. Poisson regression modelling was used to estimate mortality in the cohort.

## Results

1531 patients had documented CD4 cell counts within 3 months of diagnosis. The median age at HIV diagnosis for those under 50 and over 50 years was 33.3 (IQR 28.0-38.7) and 54.7 (IQR 51.9-60.6) respectively. 49% of the cohort had a CD4 cell count < 350 cells/mm^3^ at HIV diagnosis. The median CD4 cell count for late presenters was 211 cells/mm^3^ (IQR 83-289). The majority of the cohort were white males and homosexual. After adjustments, individuals aged over 50 were more likely to present late (OR 2.17; 95% CI: 1.51-3.11). Late presentation was less likely in calendar periods 2006-2010 and 2002-2005 compared to 1996-2001 (OR 0.62; 95% CI: 0.47-0.81 and OR 0.72; 95% CI: 0.55-0.94). Mortality rate in the cohort after 6719.80 person years (pyrs) of follow up was 15.33 per 1000 pyrs (95% CI: 12.64-18.59). After adjustments, age greater than 50 (OR 2.62; 95% CI: 1.64-4.20) and CD4 cell count <350 at diagnosis (OR 1.93; 95% CI: 1.20-3.11) remained independently associated with increased mortality.

## Conclusions

In our cohort, late presentation and mortality are decreasing; however, individuals older than 50 were more likely to present late and had a higher mortality. Initiatives to expand HIV testing in clinical and community setting should not neglect individuals aged over 50 whom are often erroneously perceived not to be at risk of HIV infection.

